# Does it matter whose opinion we seek regarding the allocation of healthcare resources? - a case study

**DOI:** 10.1186/s12913-015-1210-8

**Published:** 2015-12-18

**Authors:** K. Kolasa, T. Lewandowski

**Affiliations:** Dept of Health Economics, Collegium Medicum, Sandomierska 16, 85-830 Bydgoszcz, Poland; Poznan University of Economics, Poznan, Poland

## Abstract

**Background:**

Societal preferences have to be taken into consideration to ensure difficult healthcare decisions are legitimate and acceptable. It has been interesting to ascertain whether attitudes towards the principles of public healthcare resources allocation are homogenous. In particular, it has been thought provoking to ask whether advancement in medical technologies, and growing accessibility issues due to scarcity of healthcare resources, have influenced the beliefs of the general public with regard to allocative principles in recent years. The objective of this study was to compare preferences towards the distribution of healthcare resources between younger and older members of society.

**Methods:**

Discrete choice experiments using the equivalence of numbers technique and the social welfare function were conducted in Poland. Public preferences towards disease severity, and potential to benefit, as well as aversion to inequity, were elicited. In order to ensure full understanding of questions by the older respondents, a pilot study with ten respondents aged 65+ was conducted.

**Results:**

In total, 52 adult respondents (seniors) and 45 students (juniors) were interviewed. While the first were unwilling to trade between different patients, the latter chose a higher number of individuals to compensate for the loss of ten patients with a more severe disease and a higher potential to treat everything else being equal. Juniors were more inequality averse compared to seniors as well.

**Conclusions:**

While the revealed preferences of seniors were egalitarian, juniors were more willing to differentiate between disease severity and potential to benefit. Differences in opinion between juniors and seniors should be considered in open dialogue regarding healthcare rationing. Insight into the preferences towards health maximization of the former group and the egalitarian beliefs of the latter group could be useful for decision makers in the search for public acceptance of allocation of scarce healthcare resources.

**Electronic supplementary material:**

The online version of this article (doi:10.1186/s12913-015-1210-8) contains supplementary material, which is available to authorized users.

## Background

Life expectancy at birth has increased by roughly 10 years across OECD countries in the last 40 years. In 1950, the proportion of Europeans aged 60 plus was 15 % and it had increased to 26 % in 2010 [1]. In addition, the growing accessibility of medical information has certainly raised the expectations of patients with regard to the various treatment options. Even though health expenditure per capita has been growing at 4 % annually, this has not been enough to meet all healthcare needs [1].

The contrast between growing needs of patients and scarcity of financial resources lead to conflict between two objectives of the healthcare system: effectiveness and equity. While the first implies health maximization within given budget limits, the second introduces non-economic arguments into the decision making process. If effectiveness is chosen over equity, decision makers tend to reimburse medical technologies offering the greatest health gains for the greatest number of individuals within given financial limits. If equity over effectiveness is chosen, patients with a severe disease or lack of alternative treatment options are primarily considered. The difficulties of meeting the needs of two contradictory objectives of a health care system may create some challenges for decision makers and potentially some tensions between them and the general public.

Societal concerns have to be taken into consideration to ensure difficult healthcare decisions are legitimate and acceptable. Exploring societal preferences can inform future healthcare policy decisions and can reveal any differences between government approaches and public priorities. Only when communication between decision makers and the general public is based on mutual trust and understanding will the implementation of healthcare rationing receive public acceptance. As Guido and Bobbitt argued, allocation of healthcare resources should be conducted in a specifically established process where restrictions are imposed upon itself collectively by society. They define it as an informed democratic consensus model where consensus is an outcome of rational, moral and scientific deliberation. The authors call for ’moral foundations of social collaboration’ [2].

In order to obtain successful communication between the general public and decision makers, it is first necessary to ensure that society fully understands the consequences of limited public funding and the opportunity costs of each reimbursement decision. Therefore health policy makers need to provide public opinion with the appropriate insight into what kind of budget challenges arise in the public funding distribution process. Unless society understands the perspective of the health policy makers and the latter understand society’s preferences, decisions about who to prioritize and who to deny access to treatment cannot be regarded as democratic. It is commonly believed that public preferences have been borne in specific historical contexts and cultural environments. However, a question should be raised about whether growing healthcare needs and the increasing pressure on public budgets lead to the general public recognizing that it is impossible to secure access to all innovative treatments for all patients. In particular, it was interesting to ascertain whether the need for healthcare rationing is becoming more acceptable and consequently whether attitudes towards the principles of public healthcare resource allocation have changed over time.

The thermostatic model of policy responsiveness could be mentioned while deliberating on how open dialogue in the healthcare sector can increase awareness of the need for healthcare rationing. The model is built on the assumption that public opinion and the policy making process constantly adjust and readjust to each other over time. Execution of policy action releases public tension while lack of policy action triggers it [3]. The thermostatic model of policy responsiveness indicates therefore that the initiation of open debate can minimize the risk of public tension in the process of healthcare rationing and can even influence societal preferences towards allocation criteria.

The Polish jurisdiction was chosen for the purpose of this study. Given the historical shift from communism to democracy, Poland offers a unique setting to study the evolution of societal preferences towards the distribution of healthcare resources. Along with the transformation of the political system, the socialist principle of free access to the healthcare system was replaced by the free market economy principle of effectiveness. Nevertheless the conflict between both principles remains. While the Polish Constitution still refers to the requirement of equity in access to the public healthcare sector, the pricing and reimbursement regulations introduce the health maximization principle by referring to the explicit cost effectiveness threshold.

As it is impossible to retrospectively track the evolution of societal preferences towards the distribution of financial resources in the healthcare sector, the objective of this study was to compare allocative attitudes between younger and older groups of society. It was believed that the insight into potential differences in the preferences towards allocation principles across age groups could help decision makers to meet the expectations of a heterogeneous population more effectively. The underlying hypothesis was that the need for healthcare rationing is more acceptable to the younger generations than senior generations. Two research questions were posed. The first was to investigate whether egalitarian preferences introduced during the socialist era were more common among seniors than juniors in Poland. The second was to ask whether the younger generation were more willing to accept healthcare rationing in the healthcare sector compared to the other group.

## Methods

A discrete choice experiment based on preference-based evaluation techniques using face-to-face interviews was designed. A written informed consent for participation in the study was obtained from all participants. Since the study was not classified as a medical research, no ethical approval was required by the Polish pharmaceutical Act of 6 September, 2001.

In order to ensure the senior respondents understood the questions, a pilot study comprising individual interviews was conducted with 10 retired 65+ respondents first. As a result a final questionnaire was constructed. The study questionnaire consisted of three parts. All of them were discrete choice experiments. The equivalence of numbers technique (patient trade off, PTO) was adopted to elicit disease severity and potential to benefit weights in the first and second parts respectively [4]. In the last one, a social welfare function approach was taken to calculate the inequality aversion parameter.

The concept of utility was introduced with the support of a visual analog scale for the PTO experiments. EQ5D domains were adopted to describe the health states associated with the specific utility levels used in the experiments [5]. Following the approach adopted by other researchers, the preferences of a median respondent were estimated across all experiments [6].

In addition to socio-demographic characteristics, questions regarding past experiences of the healthcare system were included in the questionnaire.

**Fig. 1:**
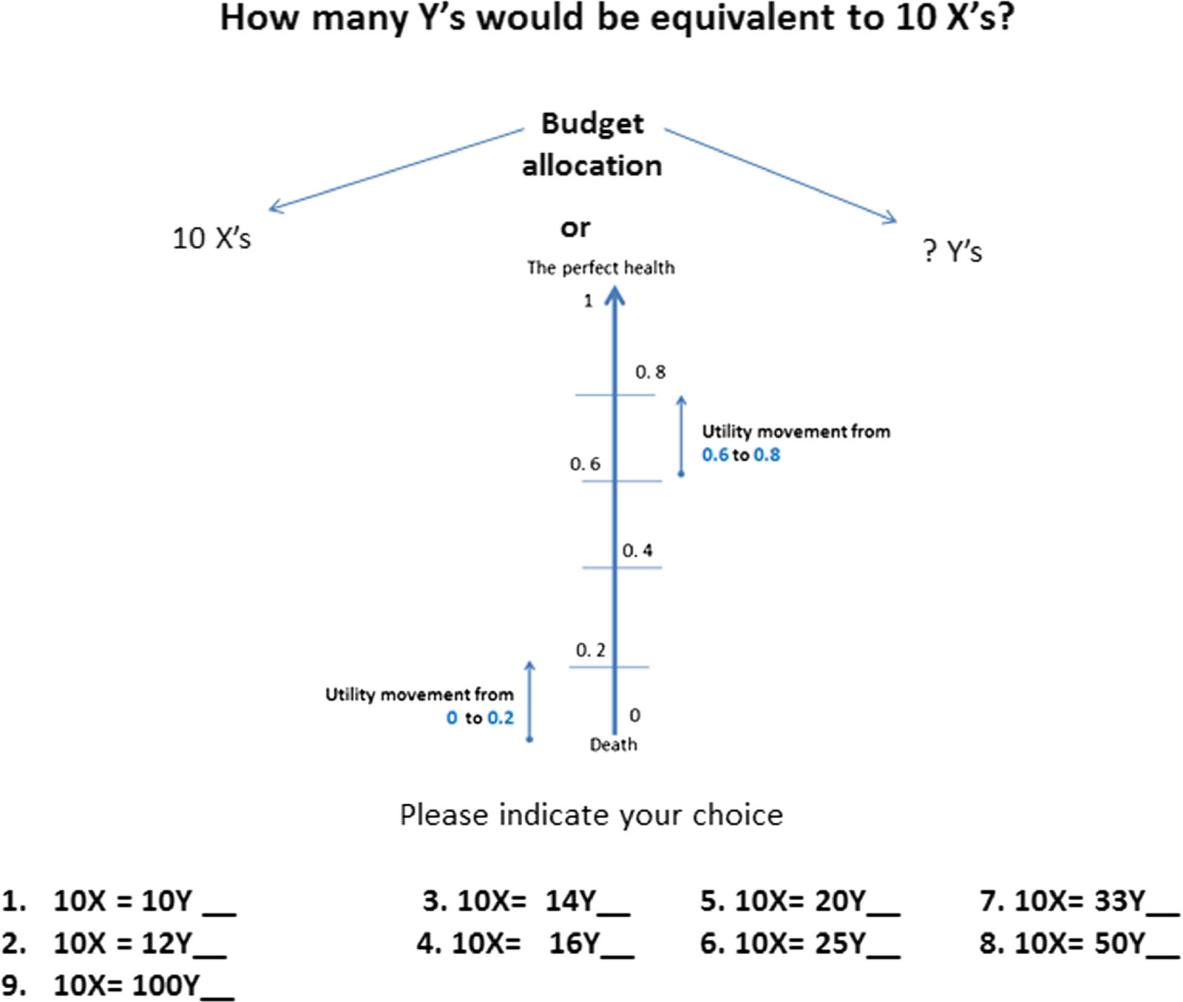
Example of PTO question

The first experiment consisted of four scenarios to assess preferences towards disease severity. In similarity with other studies which adopted the same framework, a respondent answered a question about the number of Y to be treated to make it equivalent to 10 X’s with a worse disease severity [7]. Across all scenarios, the health gain was assumed to be the same for both X and Y. The X’s baseline utility was always set to 0 and it changed by two increments starting from 0.2 with each scenario for Y. Every time a respondent had to choose one of nine options representing the number of Y being equivalent to 10 X’s i.e.10, 12, 14, 16, 20, 25, 33, 50 or 100. If a respondent chose more than 10 Y’s (i.e. favored treatment for more patients with less severe baseline utility to compensate for the lack of treatment for 10X with worse severe baseline utility), he/she was labeled as a supporter of disease severity. If the respondent expressed egalitarian preferences and was unwilling to trade Y against X, he/she was classed as an opponent. The example of PTO question is presented in Fig. 1.

Following earlier studies, a severity weight was calculated in the following way [8]:

1 $$ S{W}_j = \left({L}_x/L{M}_y\right) $$

where:

*L*_*x*_ – number of patients *X* (set to 10),

*LM*_*y*_ – number of patients *Y* (median answer),

*j* − scenario (from 1 to 4).

SW values range from 0 to 1.The higher the SW, the higher the disease severity weight. The health states not estimated in the experiment were calculated by linear extrapolation.

The second experiment consisted of three scenarios to assess preferences for capacity to benefit. In similarity to other studies which adopted the same approach, the baseline utility was set to 0.2 for both X and Y, and X had a higher health gain than Y across all scenarios [7]. While X’s health gain remained unchanged at 0.6, it varied from 0.2 to 0.6 for Y respectively. A respondent had to choose the number of Y to be treated as a compensation for the loss of 10 X with the same baseline utility but greater health gain. As with the first part, a respondent was given nine options to choose from with respect to numbers of patient Y equivalent to 10 X’s. If a respondent chose any option other than 10 Y’s, he/she was labeled as a supporter of capacity to benefit. If the respondent expressed egalitarian preferences and was unwilling to trade Y against X, he/she was classed as an opponent.

In line with earlier studies, first the *relative potential ratio* (RPR) was calculated [8]:

2 $$ \mathrm{R}\mathrm{P}\mathrm{R} = \left(U2\ \hbox{--} U1\right)\ /\ \left(1\ \hbox{--} U1\right) $$

Second, the capacity to benefit weight (*PW*) for different RPR was estimated:

3 $$ {L}_{yj} \cdot \left({U}_{2y}\hbox{--} {U}_{1y}\right) \cdot P{W}_{yj}={L}_{xj} \cdot \left({U}_{2xj}\hbox{--} {U}_{1xj}\right) \cdot P{W}_{xj}, $$

where:

*U*_1*y*_ – starting utility level for *Y*,

*U*_2*y*_ – ending utility level for *Y*,

*U*_1*x*_ − starting utility level for *X*,

*U*_2*x*_ − ending utility level for *X*,

*L*_*x*_ – number of patients *X* (set to 10),

*L*_*y*_ − number of patients *Y* (median answer),

*PW*_*xj*_ − capacity to benefit weight *X*,

*PW*_*yj*_ − capacity to benefit weight *Y*,

*j* − scenario (from 1 to 3).

*PW* increases with falling values of RPR. As before, the health states not estimated in the experiment were calculated by linear extrapolation.

The third experiment tested equity efficiency trade off. It aimed to understand the preferences of respondents towards the principle of health maximization and the reduction of health inequalities. A social welfare function (SWF) was applied as it was recognized as the most appropriate approach to test a trade-off between two conflicting objectives [9]. Following Dolan and others, a specification of constant elasticity of substitution was chosen for the purpose of this study [9, 10].

4 $$ {W}_1={\left[\upalpha {H}_a^{-r}+\upbeta {H}_b^{-r}\right]}^{\frac{1}{r}},\kern0.5em {H}_a,{H}_{b\kern0.5em \ge \kern0.5em 0,\kern0.5em \upalpha \kern0.5em +\kern0.5em \upbeta =\kern0.5em 1,\kern1em r\kern0.5em \ge \kern0.5em -1,\kern0.5em \ne \kern0.5em 0,} $$

where;

*W*_1_ - social welfare

*H*_*a*_, *H*_*b*_ - average health (*H*_*a*_ better-off, *H*_*b*_ worse-off)

λ_i_ β - weights assigned to A and B respectively, representing the societal preferences to a

given gain in well-being

r – aversion to inequality

The adaptation of SWF aimed to determine how much a program designed for the worse-off could be considered equally as valuable as a program for the better-off. More specifically, a respondent was presented with a choice between two treatment options for the better-off and worse-off. Everything else being equal, there was a difference of 5 years in average life expectancy between both groups. In each of five scenarios, a respondent had to choose between program X which added equally 4 years of additional life to both groups and program Y which favored only the worse-off by adding 8 years in the first scenario and one year less in each consequent scenario. If a respondent selected program X at the first choice, the interview was finished. Otherwise an indifference point was established between the last point at which X was chosen and the first point at which Y was selected.

Following Dolan and colleagues, the results of the third experiment were used to define the slope of SWF. Marginal rate of substitution (MRS) implies the weight given to a unit of health to one group relative to another [10].

5 $$ \mathrm{M}\mathrm{R}\mathrm{S}\ {=}^{\frac{\upalpha}{1-\upalpha}{\left(\frac{H_b}{H_a}\right)}^{1+r}} $$

The parameter r, which measures the degree of aversion to inequality, was calculated as well.

6 $$ r\approx \frac{ \log \left[\left({H}_B(X)-{H}_B(Y)\right)/\left({H}_A(Y)-{H}_A(X)\right)\right]- \log \left[\upalpha /\left(1-\upalpha \right)\right]}{ \log \left[\left({H}_B(X)+{H}_B(Y)\right)/\left({H}_A(X)+{H}_A(Y)\right)\right]}-1. $$

Following the assumption of constant elasticity of substitution (CES); α = 1-α. If r = 0, no aversion to inequality was assumed. If r > −1, there was an aversion to inequality [10]. It implied diminishing MRS between both groups.

## Results

In total 97 study participants were recruited, of which 52 were 65+ (seniors) and 45 were students under the age of 24 (juniors). The seniors were recruited to the study from one city with fewer inhabitants than 100 000, and two cities below and two cities above 250 000 inhabitants. The juniors were recruited from Warsaw Medical University’s Department of Pharmacy. The interviews were conducted between 1^st^ April and 25^th^ June 2014.

While the majority of seniors were aged between 65 and 70, more than 80 % of juniors were below 24 years old. In both groups, females prevailed. The majority lived in cities with more than 50 000 inhabitants (Additional file 1: Table S1). While more than 80 % of seniors had used healthcare services in the last month, less than one in five juniors reported any medical visits. Among seniors, problems with access to the healthcare system were less common compared to the junior group.

**Fig. 2:**
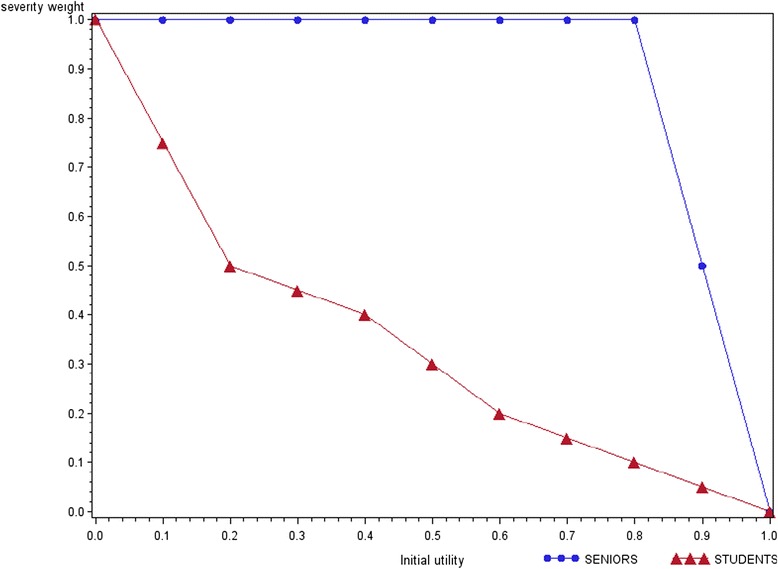
Disease severity (Experiment 1)

The first experiment revealed that the majority of seniors did not trade patients less severely ill (Y) against those who were more severely ill (X). At the same time, most juniors preferred to treat more Y as a compensation for the loss of 10 X. The differences between the number of approvers and opponents of disease severity in both groups were statistically significant across all scenarios of the experiment (Table 1). While the median senior respondent always selected 10 Y patients, the equivalent number for the median junior respondent varied from 20 to 100 in the first and last scenarios respectively (Additional file 1: Table S2). As illustrated in Fig. 2, the disease severity weight decreased with increasing utility at baseline for the younger respondents, but remained constant throughout the experiment for senior respondents.

**Table 1:** Preferences towards disease severity as an allocation criteria (Experiment 1)

	Seniors	Students	Total
approver	19 20 %	40 41 %	59 61 %
opponent	33 34 %	5 5 %	38 39 %
Total	52 54 %	45 46 %	97 100 %
X^2^ = 27.7455		*p*-value < .0001

**Fig. 3:**
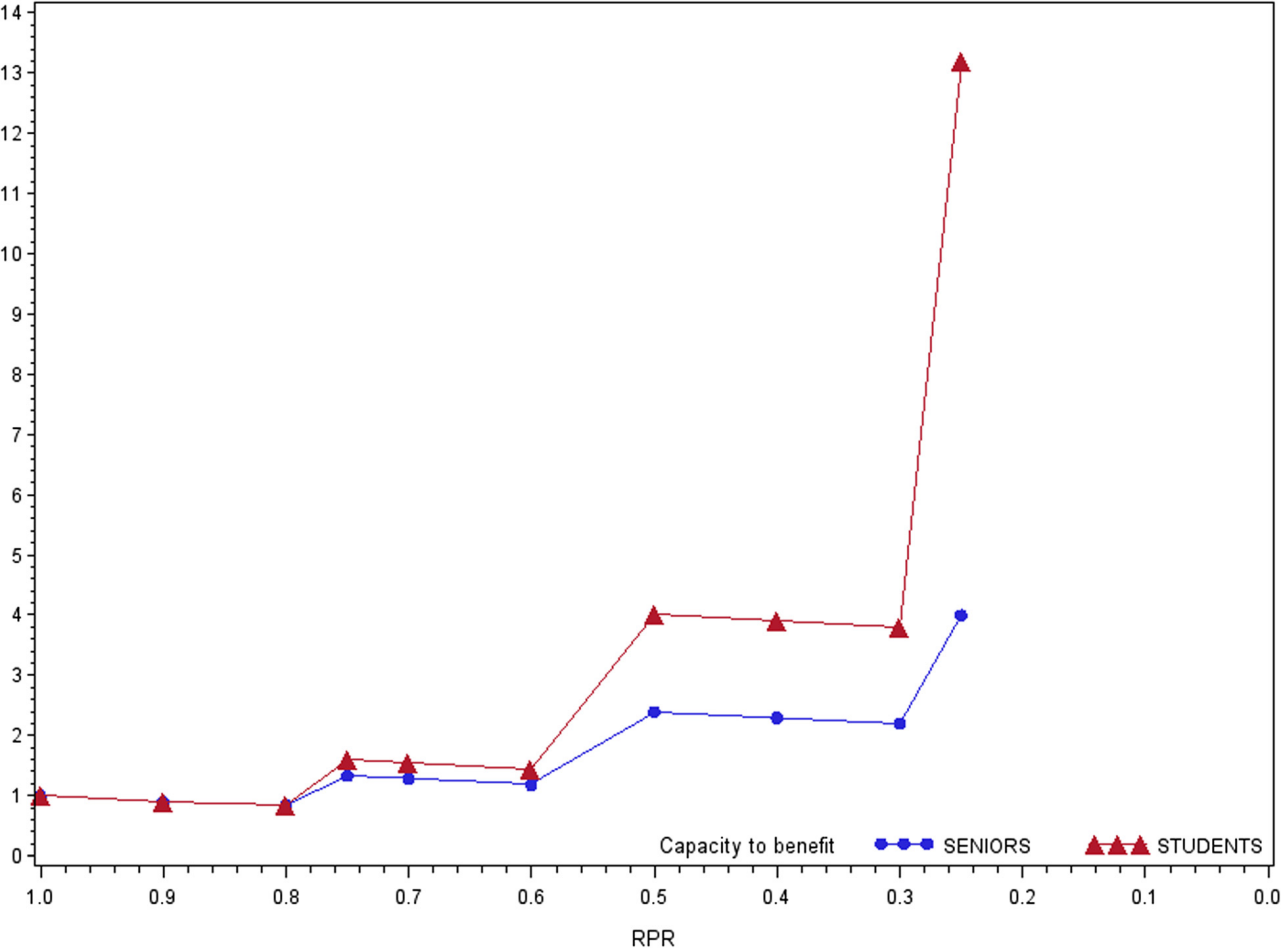
Potential to benefit weights (Experiment 2)

In the second experiment, both juniors and seniors tended to select more patients with smaller health gain (Y) as a compensation for the loss of ten individuals with a bigger potential to benefit (X). In contrast to the seniors, there were however more approvers among the junior respondents (Table 2). The median junior tended to select more Y each time as the difference in a treatment effect between X and Y diminished in the following scenarios. No specific trends for a median senior could be distinguished (Additional file 1: Table S3). In the case of both the young and senior groups, the capacity to benefit weights increased with relative potential ratio (Fig. 3). The curve was however steeper in the first group.

**Table 2:** Preferences towards potential to benefit as an allocation criteria (Experiment 2)

	Seniors	Students	Total
approver	29 30 %	40 41 %	69 71 %
opponent	23 24 %	5 5 %	28 29 %
Suma	52 54 %	45 46 %	97 100 %
X^2^ = 12.8870		*p*-value 0.0003

The results of the third experiment revealed that the median junior respondent was inequality averse (Table 3). S/he was indifferent about better- and worse-off patients living on average to be 69 and 59, respectively (i.e. the outcome of Program X) and these groups living to be 65 and 61, respectively (i.e. the outcome of Program Y). This implies that an additional life year to the worse off is valued twice as much as for the better off. At the same time the median senior respondent did not trade additional life years between both groups, choosing instead program X at the initial stage.

**Table 3:** Preferences towards inequality aversion (Experiment 3)

	Scenario	Additional life years				Indifference points				Number of switched	Inequality aversion parameter	Relative weight
		Program X		Program Y		Program X		Program Y				
		A	B	A	B	A	B	A	B			
Seniors	**1**	**4**	**4**	**0**	**8**	**69**	**59**		**63**	**21**	**−1**	**1**
	2	4	4	0	7	69	59	65	62	1	1,8191	1,60149
	2,5	4	4	0	6,5	69	59	65	61,5	0	3,4261	2,09466
	3	4	4	0	6	69	59	65	61	0	5,2815	2,85578
	4	4	4	0	5	69	59	65	60	2	10,6774	7,03398
	5	4	4	0	4	69	59	65	59	11	.	.
Juniors	1	4	4	0	8	69	59	65	63	14	−1	1
	2	4	4	0	7	69	59	65	62	2	1,8191	1,60149
	***2,5***	***4***	***4***	***0***	***6,5***	***69***	***59***	***65***	***61,5***	***0***	***3,4261***	***2,0947***
	3	4	4	0	6	69	59	65	61	11	5,2815	2,85578
	4	4	4	0	5	69	59	65	60	12	10,6774	7,03398
	5	4	4	0	4	69	59	65	59	3	.	.

Median responder in bold

Neither past experience with healthcare services nor problems with access to healthcare providers has been associated with the response rate across all three experiments (Additional file 1: Table S4).

## Discussion

The objective of this research was to verify whether society’s preferences with respect to the principles of healthcare financing allocation differed between junior and senior respondents. To the best of our knowledge this study was the first attempt to compare attitudes towards equity and effectiveness across age groups. Poland was selected for the purpose of this research. The up to date studies on societal preferences which were performed with respect to resource allocation in the healthcare sector revealed a prevalence of egalitarian attitudes among Poles. Hence it was thought provoking to ask whether it was a relic of the previous socialist system and was only dominant among the elderly, or if it is a common feature of Polish society and as such no differences between younger and older groups could be distinguished. The underlying hypothesis to be tested was whether the need for healthcare rationing is more acceptable to younger than older generations. In particular, it was to test whether public attitudes towards such allocation criteria as capacity to benefit and disease severity depend on the age of the respondent.

The results revealed some interesting findings. First of all, there were significant differences between seniors and juniors regarding their preferences towards the importance of disease severity as an allocation criterion. While the first did not differentiate between patients based on their baseline health, the majority of students took disease severity into consideration in their decision making process. When the beginning health of Y was eight times better than that of X, the median junior respondent selected a ten times higher number of Y as an equivalent number for 10X than the median senior respondent did. The disease severity weight decreased with increased baseline utility for the junior group, but was constant for the senior group.

Secondly, there were differences between the junior and senior groups with respect to inequality aversion tested in the frame of social welfare function as well. Again senior respondents were unwilling to trade additional life years gained between X and Y based on their severity of disease. The opposite was true for the junior group. While the median respondent assigned the same value to the additional life year for both groups irrespective of their life expectancy, the median junior respondent valued the health gain of the worse-off twice as much as the one for the better-off.

Finally, the study revealed strong support for capacity to benefit as an allocation criteria among junior respondents. The bigger the health gain difference between X and Y, the more Y were selected by young respondents as the equivalence of 10 X. Consequently, the potential to benefit weight increased with growing RPR. At the same time, senior respondents remained indecisive about whom to prioritize and as a consequence their potential to benefit weight changed in an inversely proportional manner to RPR.

Overall the findings confirmed both research questions. While juniors tended to differentiate between patients with regard to disease severity and potential to benefit, there was a profound lack of willingness to trade between different patient groups among seniors.

To the best of our knowledge, this study is the first attempt to compare the attitudes of juniors and seniors regarding the principles of healthcare allocation. Yet there are other studies that indicate the importance of a respondent’s characteristics in the valuation of health gain. Tsukia, for instance, discovered that the age of a respondent does impact upon social preferences regarding healthcare resource allocation. Young respondents (average age 20.1) tended to assign decreasing relative value of health in accordance with increasing age of a recipient. On the other hand, seniors (average age 73.2) prioritized those at the productive age against others [11].

One potential explanation for the differences in attitude towards allocative criteria across the two different age groups in our study might be the economic environment in which their cultural values were developed. For example a study conducted in an Italian setting revealed that equity preferences had changed over time. Interestingly enough, the observed changes were associated with the performance of the Italian economy [12]. There are other studies that suggest that there is a relationship between economic growth and the value of health. It has been found that for each 1 % growth in income, the value of health increases by 0.5–1.5 % [13]. Although our research was limited to the comparison of preferences between two age groups, it was certainly interesting to ascertain whether the significant economic growth in recent decades in Poland has played any role in the differences in attitude revealed between younger and older people.

An interesting finding of our study is the preference of senior Poles towards equal distribution of healthcare resources irrespective of patient characteristics. The egalitarian preferences revealed in this study are consistent with the results of other research. Similar findings came out in the panel study DIAGNOZA conducted on a group of 12 355 households in 2013. 7 out of 10 respondents were in favor of equal rights for everybody and of minimizing differences in economic status across society [14].

There are various explanations why individuals are in favor of an egalitarian approach. Some experts claim that it might be related to self-interest. If people are not confident in their future economic and health status, they might be in favor of equal distribution, which would ensure their access to the healthcare system irrespective of their financial situation. Alternative theory indicates that altruistic preferences make people favor egalitarianism. This is because healthcare services are perceived as a public good and free access to them improves people’s well-being [15].

Our study is not however free from limitations. Firstly, both the small sample size and specific socio-demographic profile of respondents requires caution to be exercised when making causal inferences in the observed associations. It should be especially underlined that seniors and juniors were not only different with respect to age but also other characteristics such as their place of residence and level of education. Hence, our results could be biased by the heterogeneity of the socio-demographic profiles of both groups as well.

Secondly, as is the case for the majority of discrete choice experiments, the way the decision problem was framed might have influenced the findings [16]. For example, Pinto and colleagues indicated that the choice of a hypothetical number of patients in each scenario of PTO experiment might impact the results [17]. Although we followed Nord and K. Wittrup-Jensen who used ten patients in their experiments, some researchers adopted a different approach with a higher number of hypothetical cases [18, 19].

Thirdly, any study such as ours bears the risk of response bias related to excessive cognitive burden, given that a respondent was asked to consider herself/himself as a decision maker. It is especially critical in case individual opinions diverge from general preferences. If so, a respondent may be unwilling to answer according to his own beliefs and instead adjust his response to the most common behavior. Miller calls it a Sunday-BEST attitude [20]. In a similar fashion, our study results could be affected by reference point bias [21]. According to this theory, a respondent refers to previously made choices in his answers. A similar bias may be introduced as the result of an anchoring effect which occurs when a respondent’s answers are influenced by some arbitrary value related to his past experience [22].

Fourthly, in addition to the above mentioned limitations, one can claim that the generalizability of our study findings is limited. There are reasons to believe that public attitudes towards allocation criteria are settings-specific. While certain restrictions in access to the healthcare sector can be approvable in a given country, they might be rejected by public opinion in another one. This is due to the fact that societal preferences are associated with the culture and sets of values born in the historical context. For example the Internet based discrete choice simulation conducted among 800 participants in South Korea found that disease severity and the socio-demographic characteristics of patients are to be considered the most important principles in the decision making process of the healthcare sector. Health maximization was identified as the second most important criteria [23]. Meanwhile, a study conducted on 3669 respondents from the UK revealed general support for maximizing Quality Adjusted Life Years (QALY) gains and disfavor for disease severity defined by reduced health-related quality of life (HRQOL) before treatment. At the same time, another study conducted in the UK found favorable preferences for disease severity defined by the life expectancy without the treatment [24].

Fifthly, in addition to the efficiency and equity, there are other key criteria that should be utilized in the decision making process in the healthcare sector but were omitted from the scope of this study. Among them, key features of the organization of a healthcare system, such as reimbursement methods of medical procedures, healthcare services utilization patterns and others should be taken into consideration. Therefore in order to develop a full list of allocative criteria, these other considerations should also be taken into account.

Finally, it is likely that the results of our study could have been different if we had allowed the socio-demographic profile of the patients in question to vary. There is mounting evidence which indicates that not only does the age of a respondent impact upon his/her attitudes towards allocation criteria, but also that the age of the patient in question has an impact on the differential valuation of health gains as well. The treatment of a working population is generally considered more important compared to seniors [11]. For example, Maureen Cropper and colleagues found that on average averting the death of one twenty-year-old was considered to be of equal priority to averting the death of eight sixty-year-olds [25]. Both Cropper and colleagues and Tsuchiya and colleagues found a general preference in favor of productive age [25, 26] It has to be mentioned however that there is a German study which did not find preferences for any age groups in the resource allocation decision making process except for children [27]. In addition to the age of the patient, the literature uncovers a broad range of other attributes across which the value of health gain may be expected to vary [26]. The characteristics of the patient in question, such as his or her disease severity, the size of the health benefit, and their socio-economic background are amongst these attributes. The Italian study mentioned earlier indicated that singles and couples from 18 to 65 years and couples with children seem to care more for equity than elderly couples and three generation-households [28]. A Danish study found that inequity aversion, in particular aversion to another’s disadvantage, changes with other socio-demographic characteristics as well. The group of young and highly educated respondents was less inequity averse with the least marginal disutility to another’s disadvantage than other sectors of population included in the study [29].

Given the above limitations, we need to treat our results with caution. It is especially due to the small sample size and chosen experimental framework that our study should be regarded as a pilot one. Nevertheless, we strongly believe that it contributes to the current state of knowledge regarding societal preferences towards allocation criteria in the healthcare sector. It provides sound rationale for future research on socio-demographic differences in public preferences towards allocation principles.

## Conclusions

The contention is that there is a need for public engagement in the decision making processes regarding the allocation of limited healthcare resources. Our results indicate that it might be more difficult for seniors to accept healthcare rationing. It is believed that the egalitarian preferences among society translate into demanding attitudes towards the State. According to available public opinion surveys conducted on a representative sample of Poles, free access to the healthcare system was regarded as the State’s responsibility by 95 % of responders in 2013, which was an increase of 5 % from 1996 [28]. Therefore, health policy makers have to adopt a two-fold approach in their pursuit for optimal allocation of scarce healthcare resources. First of all, they need to acquire appropriate insight into societal attitudes towards equity efficiency trade off. Unless public preferences are followed, the moral legitimacy of tough administrative decisions cannot be achieved. Secondly, health policy makers need to constantly increase the general public’s awareness regarding how financial constraints impact upon hard choices in the healthcare sector. Any rationing decisions against the moral foundations of a given society can be recognized as a threat to the democratic rules which are defined as a set of freely self-imposed limitations.

It is hoped that our study will provide decision makers with some insight into the preferences of the general public. Given that seniors might be less prone to accept the need for healthcare rationing, decision makers could consider some additional efforts to be executed to specifically address these concerns.

The importance of open dialogue in the healthcare sector can be illustrated with an example provided by Dolan. It indicates how successful a discussion with members of the general public regarding healthcare issues can be in shaping societal preferences [30]. Based on the focus group study, Dolan indicated that attitudes towards health priorities evolve in the course of deliberation. Sixty randomly chosen patients were asked about their opinions of who to prioritize to access treatment twice at different time points. In the course of discussion and reflection, the number of respondents who wanted to discriminate against heavy drinkers, smokers and illegal drug users decreased. Interestingly enough, the preference towards prioritization of the elderly diminished as well.

As Dolan proved, we can argue that open dialogue about the need for healthcare rationing can influence societal preferences. Based on our study findings, we cannot indicate what kind of actions have to be taken to successfully communicate with younger and older groups in society respectively. Future research should therefore shed some new light on this challenge.

## Additional file

Additional file 1: (PDF 218 kb)
