# Supplementary material for: Does it matter whose opinion we seek regarding the allocation of healthcare resources? - a case study
Source: BMC Health Serv Res. 2015 Dec 18;15:564. doi: 10.1186/s12913-015-1210-8 (PMC4683956; doi:10.1186/s12913-015-1210-8)
Supplement: Additional file 1: — (PDF 218 kb) [file 12913_2015_1210_MOESM1_ESM.pdf]

Introduction to Experiment 1 and 2

- The objective of this questionnaire is to ask you about your opinion regarding the principles that should apply to the healthcare decision making process.

- The questionnaire does present only fictitious experiments that were designed solely for the purpose of this study.

- Your opinion will not influence any real situations and as such will not impact any real life decision making issues.

- Anonymity of respones will be ensured. The results will be used only for research purposes.

Introduction

Please read through the below description. Please start reading upward.
Please note that all descriptions of health state are valid throughout experiments 1 and 2.

Health state 1
Perfect health

Health state 0.8
1. No mobility problems
2. **Some problems with self care.**
3. **Being depressed.**
4. **No problems with usual activities.**
5. **No pain/discomfort**

Health state 0.6
1. Some mobility problems
2. Some problems with self care**.**
3. **Severely depressed.**
4. **No problems with usual activities.**
5. **No pain/discomfort.**

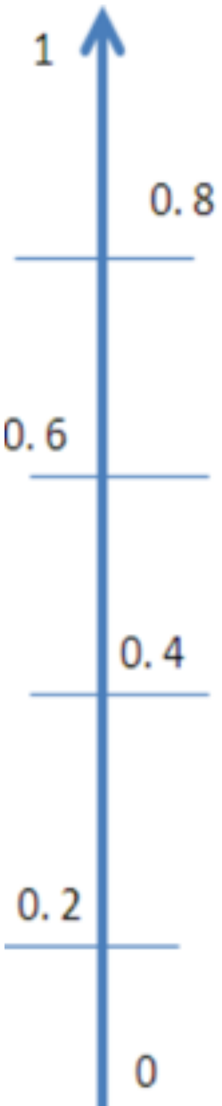

Health state 0.4
1. Some mobility problems
2. Severe problems with self care
3. Severely depressed.
1. **No problems with usual activities.**
4. **Minor pain/discomfort**

Health state 0.2
1. Some mobility problems
2. Severe problems with self care.
3. Severely depressed.
4. Severe problems with usual activities.
5. No pain/discomfort.

Health state 0.0
Death

Exercise

Please indicate your current health state

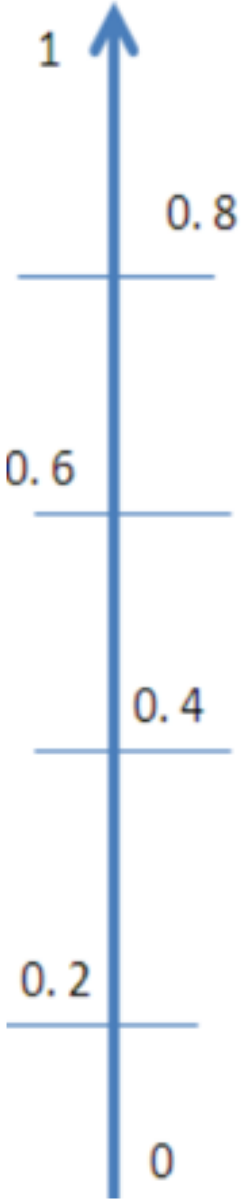

Health state 1.0
Perfect health

Health state 0.8

1. No mobility problems
2. **Some problems with self care.**
3. **Being depressed.**
4. **No problems with usual activities.**
5. **No pain/discomfort**

Health state 0.6

1. Some mobility problems
2. Some problems with self care.
3. **Severely depressed.**
4. **No problems with usual activities.**
5. **No pain/discomfort.**

Health state 0.4

1. Some mobility problems
2. Severe problems with self care
3. Severely depressed.
4. **No problems with usual activities.**
5. **Minor pain/discomfort**

Health state 0.2

1. Some mobility problems
2. Severe problems with self care.
3. Severely depressed.
4. Severe problems with usual activities.
5. No pain/discomfort.

Experiment 1

Imagine yourself in a position where you are to choose between
health care program for patients X and health care program for patients Y.

The budget costs are the same in both cases. If the total budget is to
be spent, we can treat 10 patients X. The alternative is to treat only
patients Y.

At what number of patients Y would you be indifferent
between the two programs?

Please note that arrows indicate what kind of health
improvements patients will obtain in each case.

How many patients Y should be treated to compensate for the loss opportunity of treatment for 10 patients X?

Budget

10 pts X **OR** ? pts Y

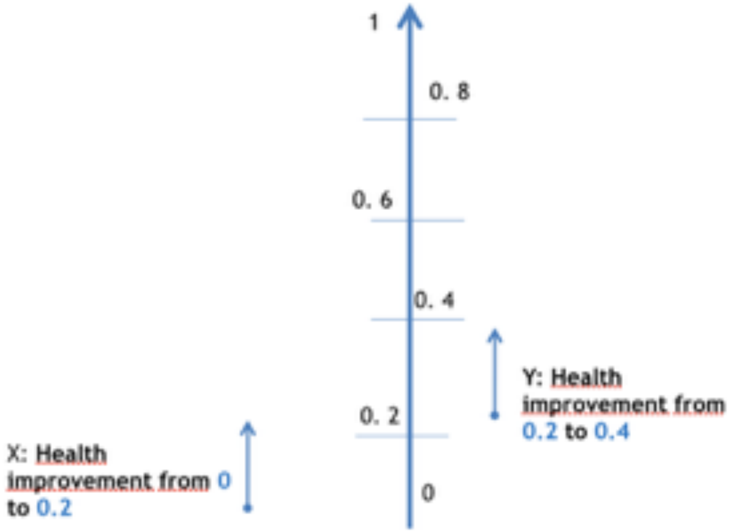

Please indicate at what number would you be indifferent between the two projects? Please cross a box to indicate your answer

1. 10X = 10Y __
2. 10X = 12Y__
3. 10X= 14Y__
4. 10X= 16Y__
5. 10X= 20Y__
6. 10X= 25Y__
7. 10X= 33Y__
8. 10X= 50Y__
9. 10X= 100Y__

How many patients Y should be treated to compensate for the loss opportunity of treatment for 10 patients X?

Budget

10 pts X **OR** ? pts Y

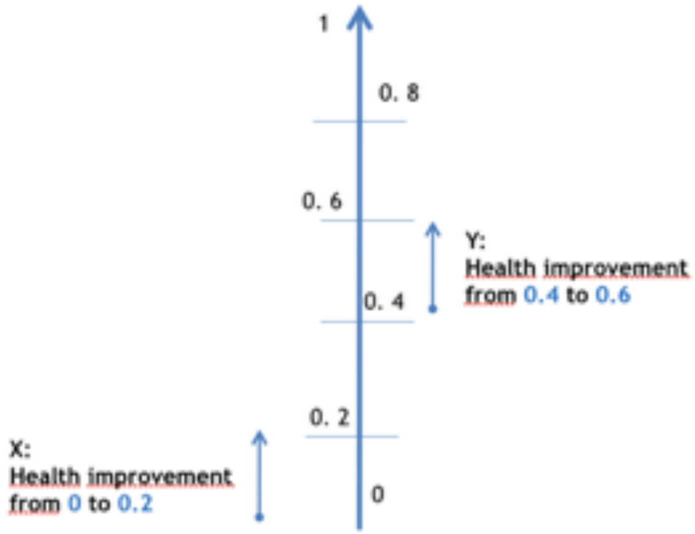

Please indicate at what number would you be indifferent between the two projects? Please cross a box to indicate your answer

1. 10X = 10Y __
2. 10X = 12Y__
3. 10X= 14Y__
4. 10X= 16Y__
5. 10X= 20Y__
6. 10X= 25Y__
7. 10X= 33Y__
8. 10X= 50Y__
9. 10X= 100Y__

How many patients Y should be treated to compensate for the loss opportunity of treatment for 10 patients X?

Budget

10 pts X **OR** ? pts Y

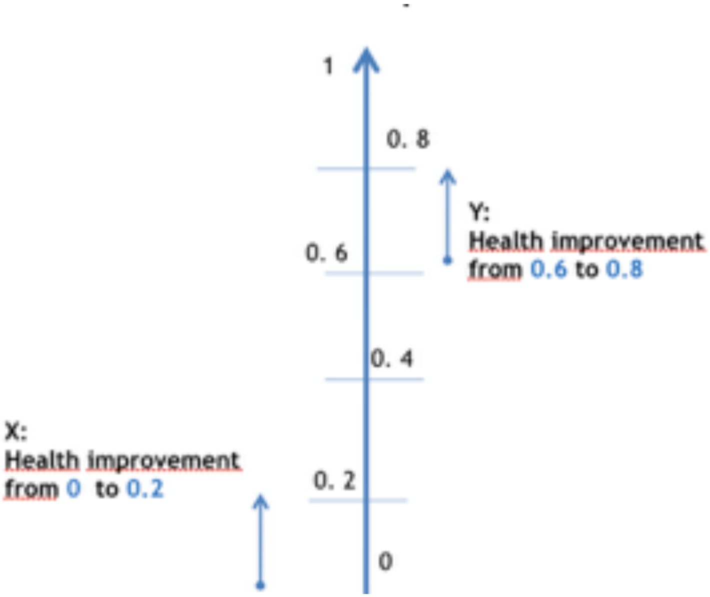

Please indicate at what number would you be indifferent between the two projects? Please cross a box to indicate your answer

1. 10X = 10Y __
2. 10X = 12Y__
3. 10X= 14Y__
4. 10X= 16Y__
5. 10X= 20Y__
6. 10X= 25Y__
7. 10X= 33Y__
8. 10X= 50Y__
9. 10X= 100Y__

How many patients Y should be treated to compensate for the loss opportunity of treatment for 10 patients X?

Budget

10 pts X — **OR** — ? pts Y

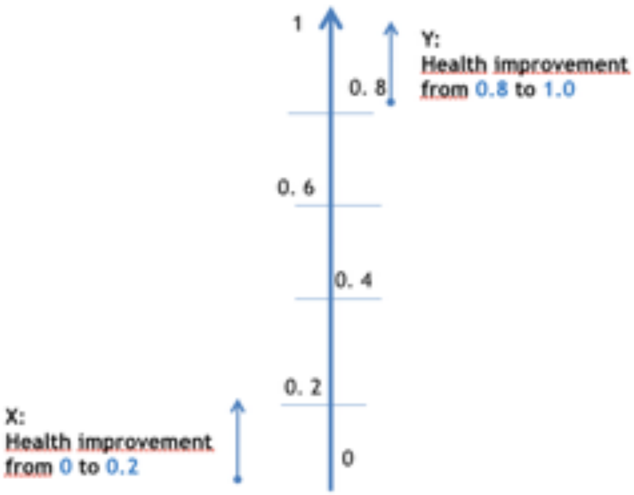

Please indicate at what number would you be indifferent between the two projects? Please cross a box to indicate your answer

1. 10X = 10Y __
2. 10X = 12Y__
3. 10X= 14Y__
4. 10X= 16Y__
5. 10X= 20Y__
6. 10X= 25Y__
7. 10X= 33Y__
8. 10X= 50Y__
9. 10X= 100Y__

Experiment 2

Imagine yourself in a position where you are to choose between
health care program for patients X and health care program for patients Y.

The budget costs are the same in both cases. If the total budget is to
be spent, we can treat 10 patients X. The alternative is to treat only
patients Y.

At what number of patients Y would you be indifferent
between the two programs?

Please note that arrows indicate what kind of health
improvements patients will obtain in each case.

How many patients Y should be treated to compensate for the loss opportunity of treatment for 10 patients X?

Budget

10 pts X — **OR** — ? pts Y

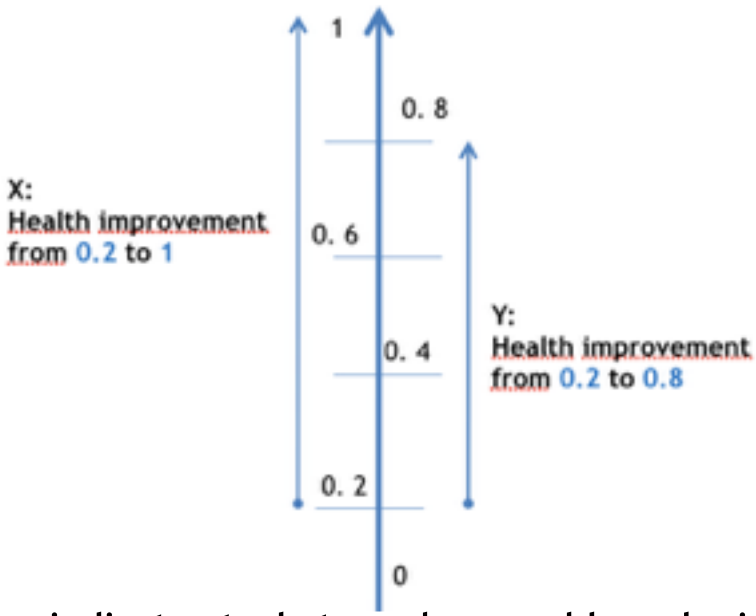

Please indicate at what number would you be indifferent between the two projects? Please cross a box to indicate your answer

1. 10X = 10Y __
2. 10X = 12Y__
3. 10X= 14Y__
4. 10X= 16Y__
5. 10X= 20Y__
6. 10X= 25Y__
7. 10X= 33Y__
8. 10X= 50Y__
9. 10X= 100Y__

How many patients Y should be treated to compensate for the loss opportunity of treatment for 10 patients X?

Budget

10 pts X

OR

? pts Y

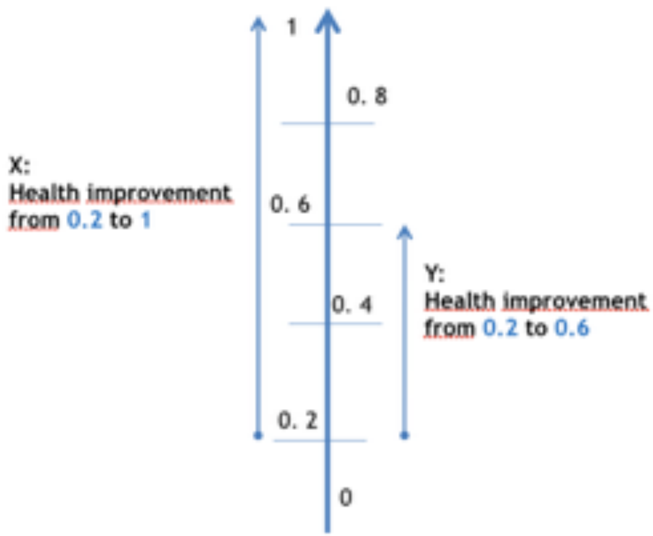

Please indicate at what number would you be indifferent between the two projects? Please cross a box to indicate your answer

1. 10X = 10Y __
2. 10X = 12Y__
3. 10X= 14Y__
4. 10X= 16Y__
5. 10X= 20Y__
6. 10X= 25Y__
7. 10X= 33Y__
8. 10X= 50Y__
9. 10X= 100Y__

How many patients Y should be treated to compensate for the loss opportunity of treatment for 10 patients X?

Budget

10 pts X **OR** ? pts Y

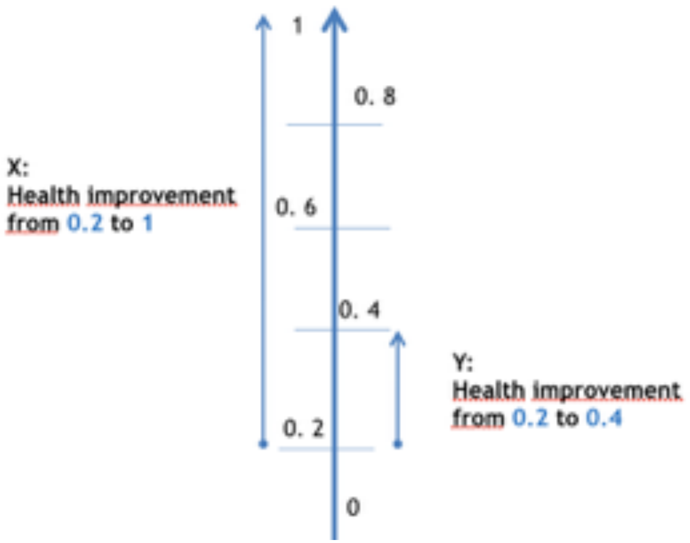

Please indicate at what number would you be indifferent between the two projects? Please cross a box to indicate your answer

1. 10X = 10Y __
2. 10X = 12Y__
3. 10X= 14Y__
4. 10X= 16Y__
5. 10X= 20Y__
6. 10X= 25Y__
7. 10X= 33Y__
8. 10X= 50Y__
9. 10X= 100Y__

Experiment 3

There are two group of patients: A i B, the same gender and at the same age (50 years old).

Group A consists of well-off individuals in good health.
Their life expectancy is additional 15 years.

Group B consists of poor individuals with health issues.
Their life expectancy is additional 5 years.

There are the same number of individuals in each group.

Imagine that you are a budget holder and have to choose one of two health programs: X or Y.

They cost the same but only one of them can be financed.

Program X extends life expectancy of both groups by equal number of years.

Program Y extends life expectancy only of individuals in Group B.

Which program do you prefer?

if response for X, stop the experiment

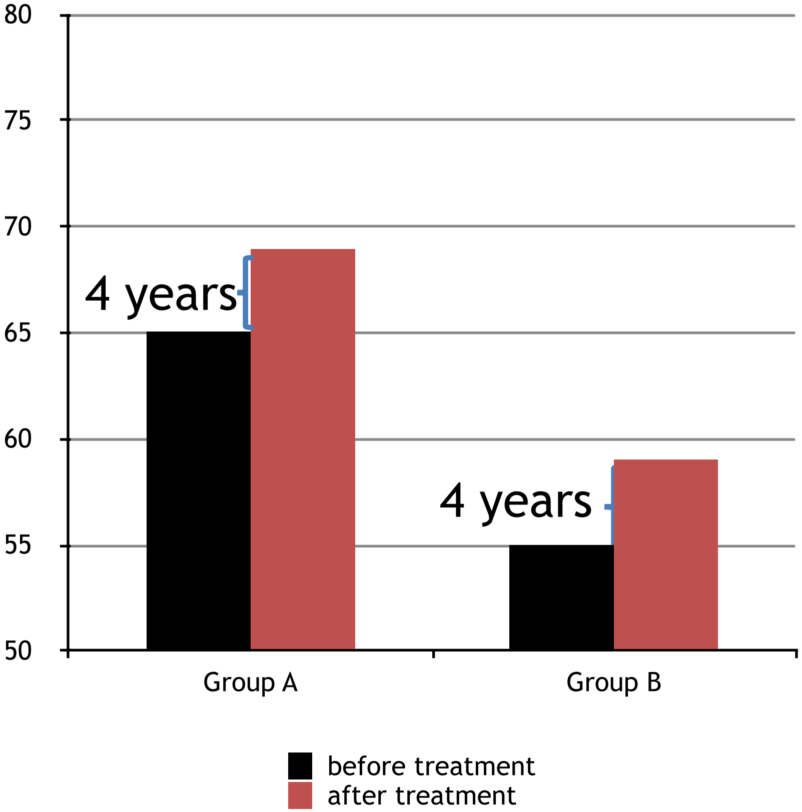

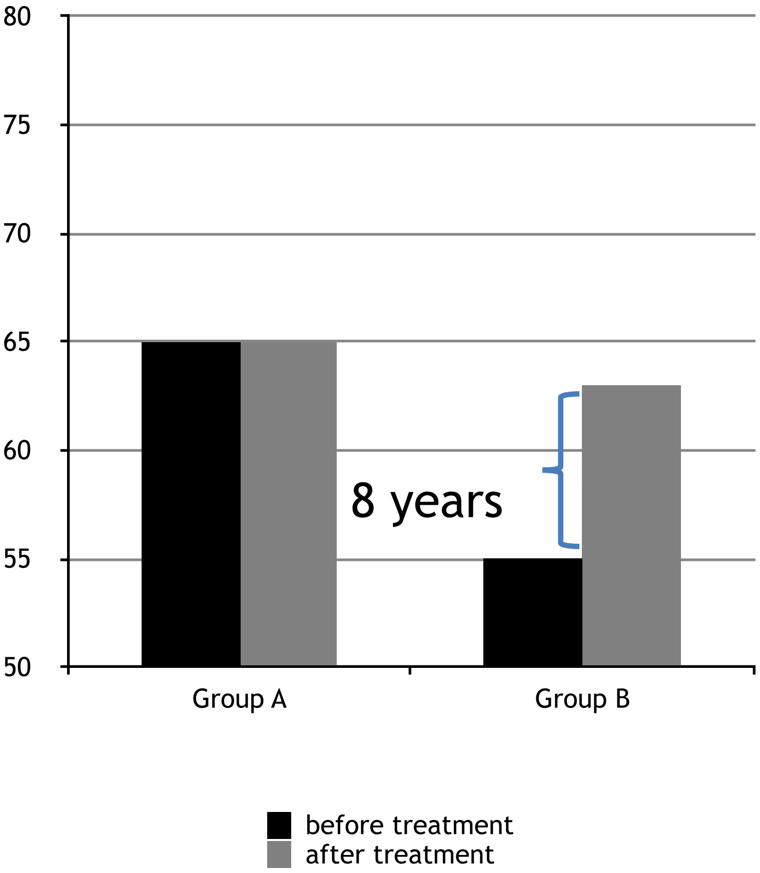

Program X

Program Y

Please indicate which program
you want to select:

Which program do you prefer?

if response for X, stop the experiment

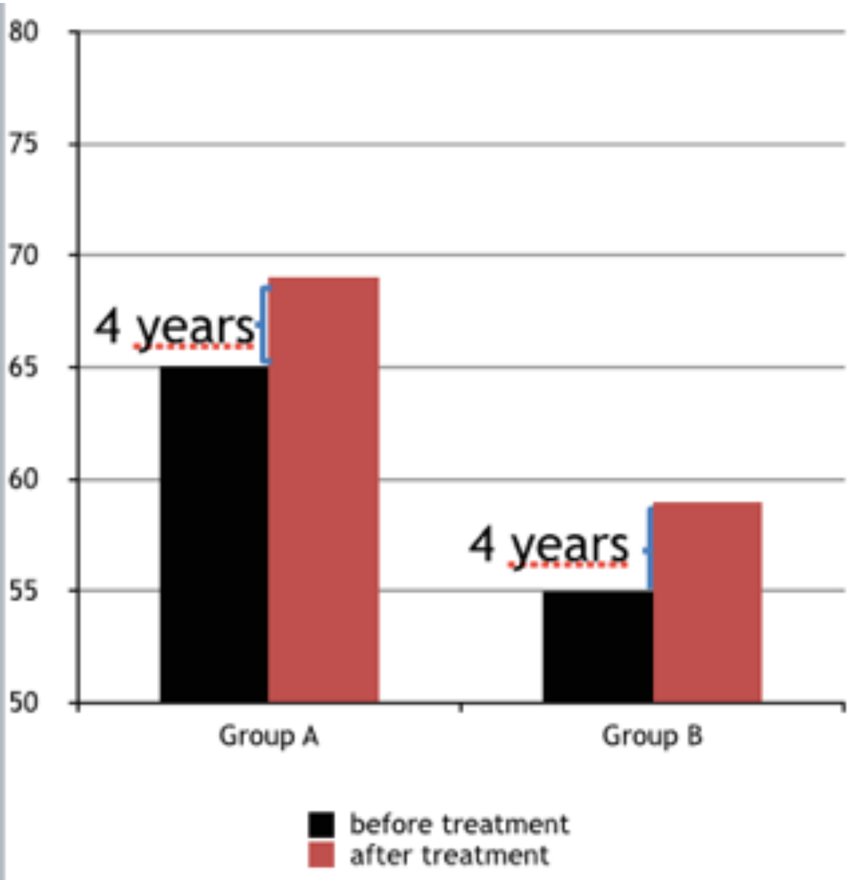

Program X

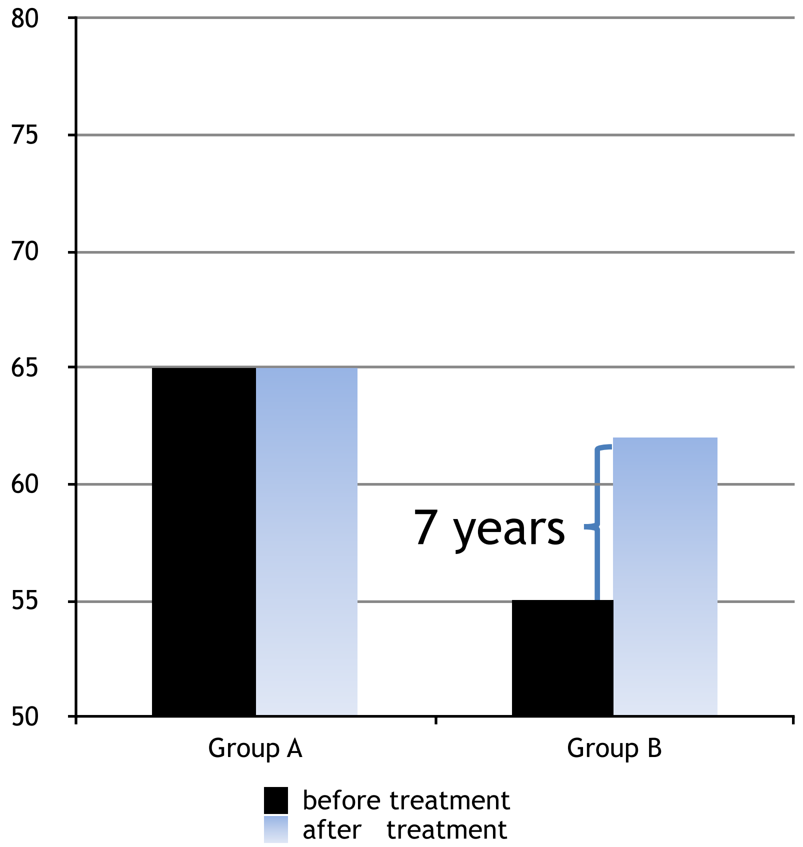

Program Y

Please indicate which program
you want to select:

Which program do you prefer?

if response for X, stop the experiment

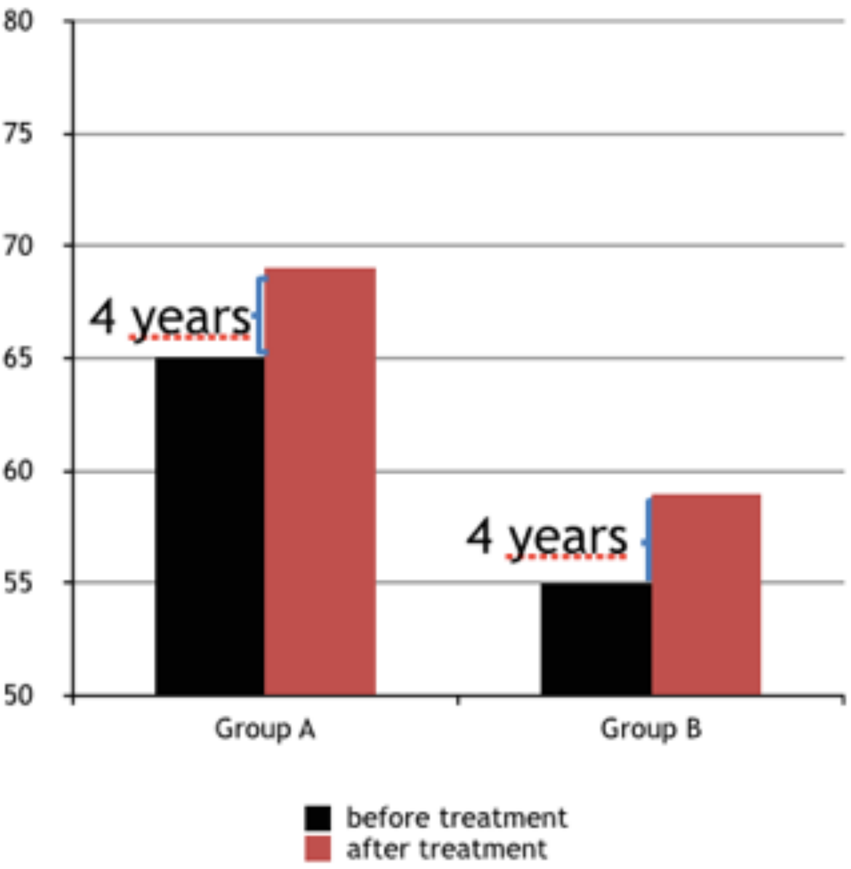

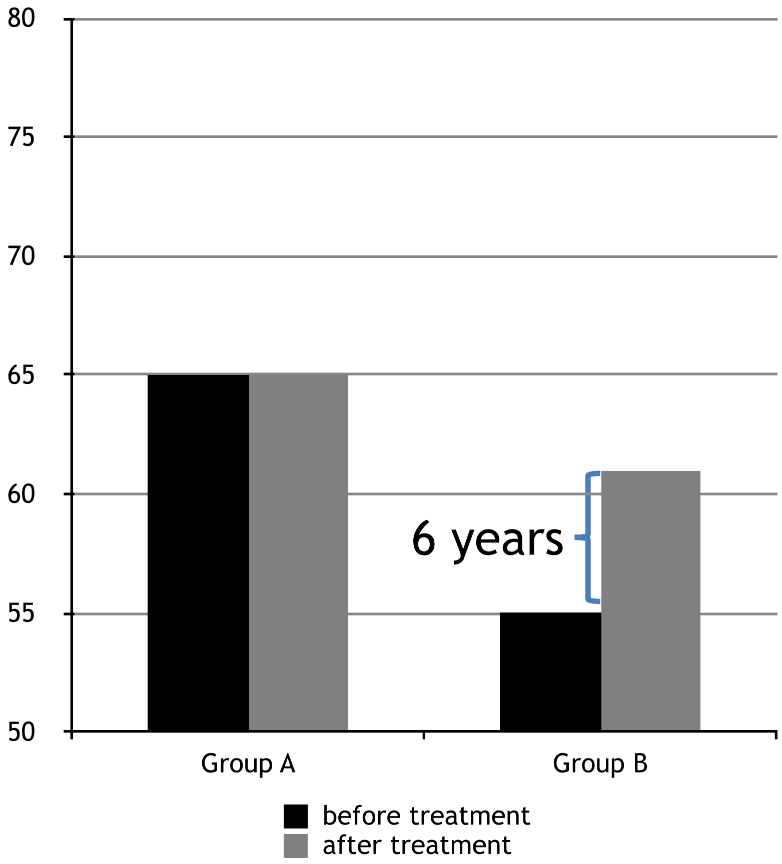

Program X

Program Y

Please indicate which program you want to select:

Which program do you prefer?

if response for X, stop the experiment

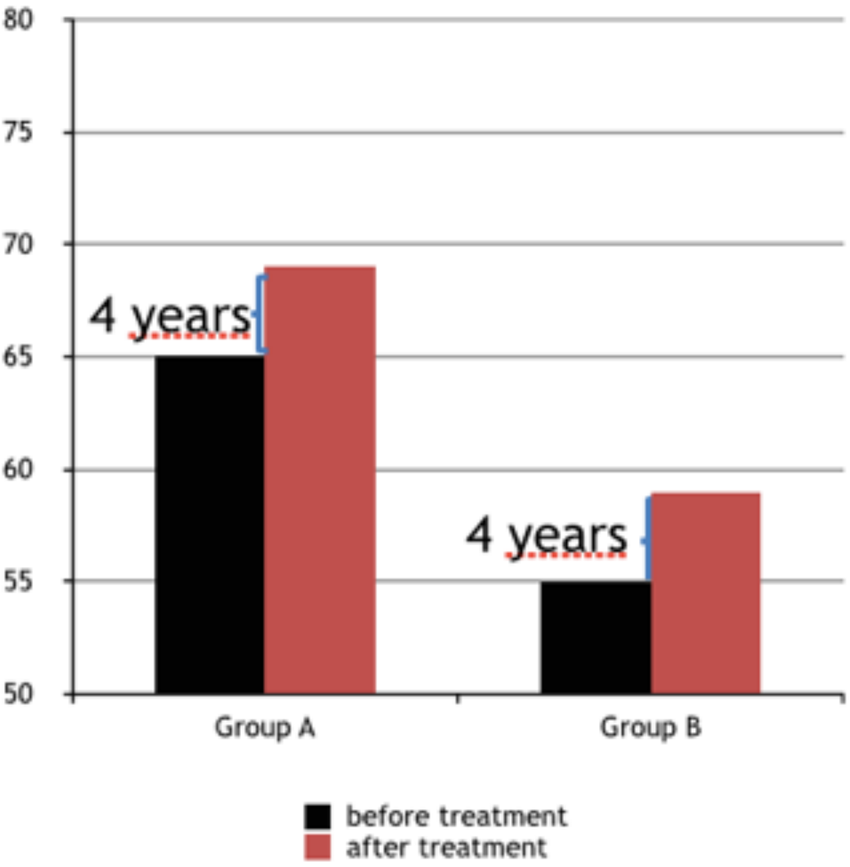

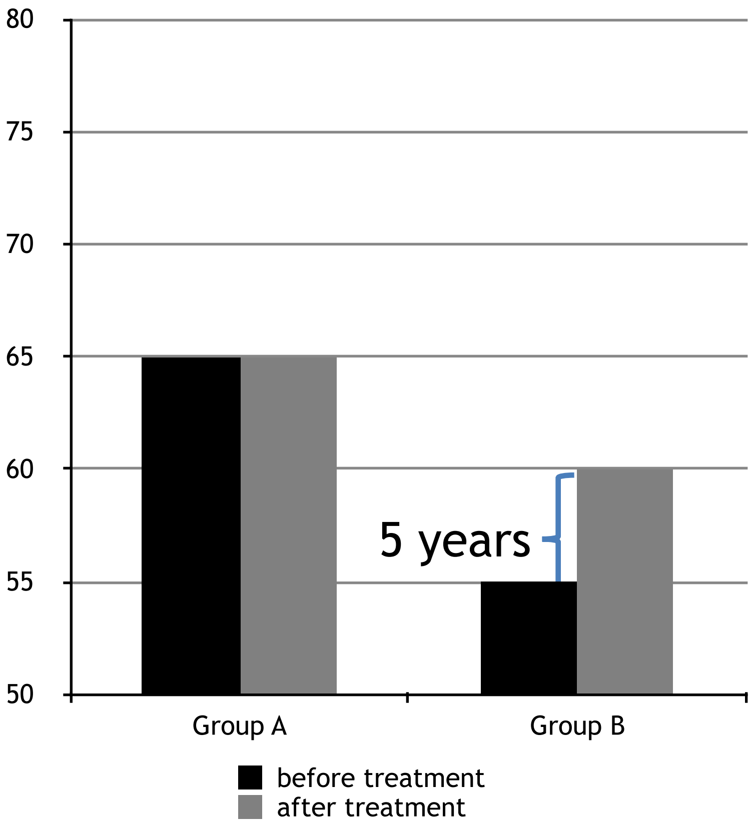

Program Y

Program X

Please indicate which program you want to select:

Which program do you prefer?

if response for X, stop the experiment

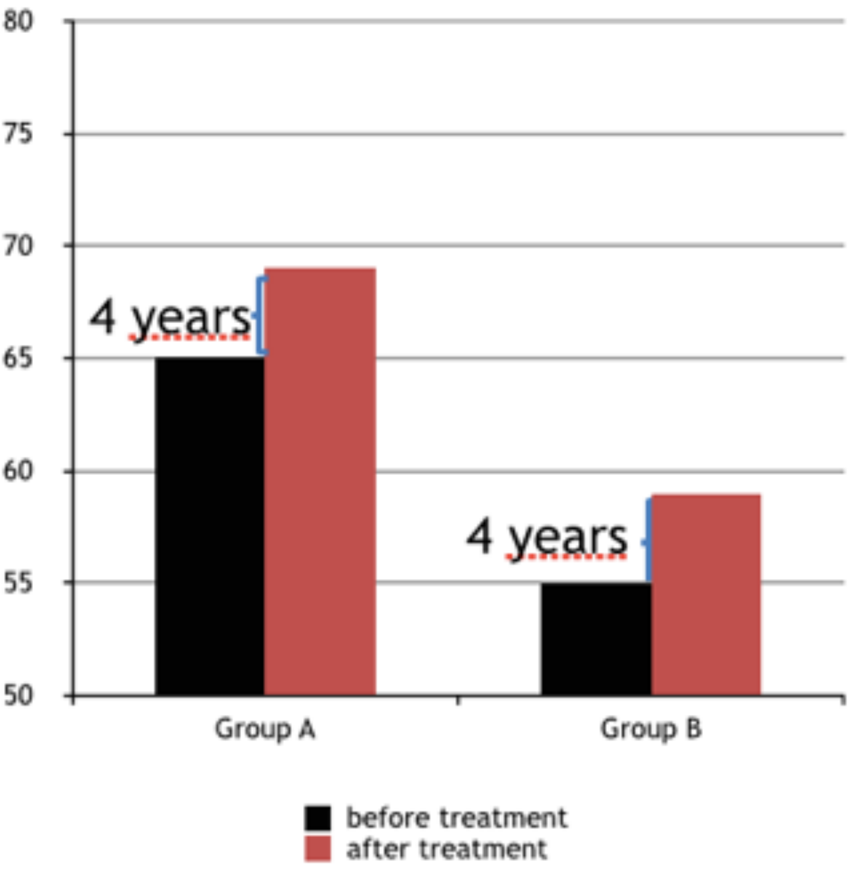

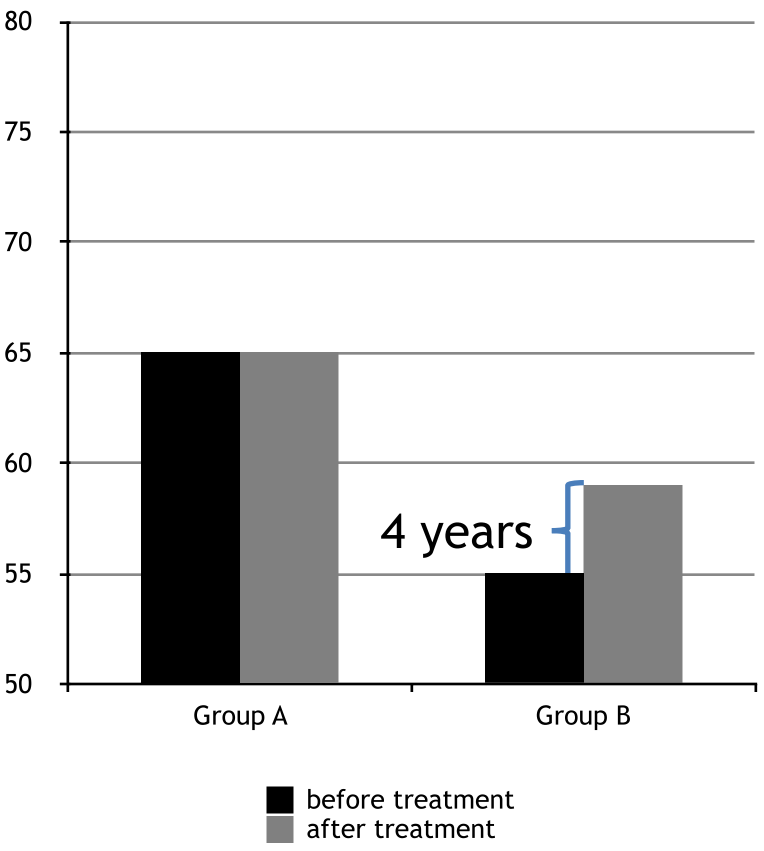

Program X

Program Y

Please indicate which program
you want to select:

Please provide the following

Age____________________

Gender____________________

Education_____________________________

Place of living. Please indicate
Countryside__________________
city (>50 000)________________
city (<50 000) _______________

Did you contact any healthcare provider in the last month?_______________________

Did you have any problems with access to healthcare services in the last 3 months? _____________,

Hereby I confirm that I allow Katarzyna Kolasa to utilize my responses for the research purposes in alignment with the law dated 29.08.1997: Dz. U. z 2002r. Nr 101, poz. 926 ze zm.

____
Signature and date

Thank you
